# Airway Basal Cells Mediate Hypoxia-Induced EMT by Increasing Ribosome Biogenesis

**DOI:** 10.3389/fphar.2021.783946

**Published:** 2021-12-09

**Authors:** Yapeng Hou, Yan Ding, Danni Du, Tong Yu, Wei Zhou, Yong Cui, Hongguang Nie

**Affiliations:** ^1^ Department of Stem Cells and Regenerative Medicine, College of Basic Medical Science, China Medical University, Shenyang, China; ^2^ Department of Anesthesiology, The First Hospital of China Medical University, Shenyang, China

**Keywords:** hypoxia, MTECs, basal cells, EMT, ribosome

## Abstract

Excessive secretion of airway mucus and fluid accumulation are the common features of many respiratory diseases, which, in turn, induce cell hypoxia in the airway epithelium, resulting in epithelial–mesenchymal transition (EMT) and ultimately fibrosis. However, the mechanisms of EMT induced by hypoxia in the airway are currently unclear. To mimic the status of edematous fluid retention in the airway, we cultured primary mouse tracheal epithelial cells (MTECs) in a liquid–liquid interface (LLI) mode after full differentiation in a classic air–liquid interface (ALI) culture system. The cell hypoxia was verified by the physical characteristics and lactate production in cultured medium as well as HIF expression in MTECs cultured by LLI mode. EMT was evidenced and mainly mediated by basal cells, supported by flow cytometry and immunofluorescence assay. The differently expressed genes of basal and other airway epithelial cells were found to be enriched in the ribosome by our analysis of an MTEC single-cell RNA sequencing data set and Myc, the global regulator of ribosome biogenesis was identified to be highly expressed in basal cells. We next separated basal cells from bulk MTECs by flow cytometry, and the real-time PCR results showed that ribosome biogenesis was significantly upregulated in basal cells, whereas the inhibition of ribosome biogenesis alleviated the phosphorylation of the mammalian target of rapamycin/AKT and abrogated hypoxia-induced EMT in MTECs. Collectively, these observations strongly suggest that basal cells in the airway epithelium may mediate the process of hypoxia-induced EMT, partly through enhancing ribosome biogenesis.

## Introduction

Oxygen is essential for human survival, deficiency in the supplement of which may lead to the injury of cell, tissue, and organ, even organism death. Hypoxic conditions are involved in cancers, stroke, and cardiovascular and chronic respiratory diseases (Semenza, 2011; Befani and Liakos, 2018). Hypersecretion of mucus in asthma/cystic fibrosis and the untimely clearance of fluid in chronic obstructive pulmonary disease can induce hypoxia in the airway epithelium (Place et al., 2017; Hou et al., 2020a). However, much is still not very clear about the relative mechanisms of hypoxia on chronic respiratory diseases.

Researchers never stopped exploring the effect of hypoxia on respiratory diseases, and various physical/chemical hypoxia models were established. Lowering the oxygen level in a CO_2_ incubator or chamber is the optimal method to induce hypoxia, which has been widely used in *in vitro* and *in vivo* experiments as an important physical method for the decrease of oxygen concentration (Polosukhin et al., 2011; Yee et al., 2016; Chen et al., 2020). According to Fick’s First Law (F = D × ∆*C*/∆*x*), the diffusion velocity (F) of a gas through a medium is positively correlated with the concentration gradient of the gas on either side of the medium (Δ*C*), and negatively correlated with the thickness of the medium (Δ*x*) (Place et al., 2017). As a replacement method for the abovementioned hypoxia incubator in our experiment, excessive culture medium can mimic the retention of fluid in the airway under pathological conditions, which also generates a hypoxic condition for cells by increasing the thickness (Δ*x*) of the medium and ultimately decreasing the diffusion velocity (F) of oxygen (Gerovac et al., 2014).

Epithelial–mesenchymal transition (EMT) is considered to be a main driving force during fibrosis, a process that loses epithelial cell identities and acquires mesenchymal cell features (Rout-Pitt et al., 2018; Bakir et al., 2020). Hypoxia can induce EMT and ultimately fibrosis formation in asthma, chronic obstructive pulmonary disease, etc., seriously affecting the patients’ respiratory function (Broytman et al., 2015; Zhou et al., 2020). Previous studies report that many pathways/factors are involved in the process of EMT, including AKT, the mammalian target of rapamycin (mTOR) signal pathway, transcription factors, epigenetic modifications, microRNAs, long noncoding RNAs, ribosomes, and so on (Karimi Roshan et al. 2019, Georgakopoulos-Soares et al. 2020). Of note, ribosome is composed of ribosome protein and RNA, and besides the typical role of cellular protein synthesis, numerous recent studies prove that ribosome proteins (S15a, S19, L14, and L22) are necessary for the EMT process (Chen et al., 2018; Wang et al., 2018; Feng et al., 2019; Liu et al., 2019; Prakash et al., 2019; Dermit et al., 2020). Furthermore, studies show that the enhanced ribosome biogenesis in the protrusions significantly strengthen the migration of cells, whereas the role of ribosome biogenesis in the airway hypoxia-induced EMT process is not fully understood (Dermit et al., 2020).

As a ribosome-associated unit, mTOR complex 2 (mTORC2) belongs to the mTOR kinase family and is composed of mTOR, mLST8, and rictor (Fu and Hall, 2020). It is reported that ribosome binds and activates mTORC2 by the association of rictor and/or mSIN1 binding to the 60S subunit of the ribosome (Zinzalla et al., 2011). Meanwhile, it is reported that the activation of mTORC2 is predominantly achieved by mTOR phosphorylated in Ser2481 (Copp et al., 2009). Recent studies show that ribosome biogenesis fuels EMT by increasing the recruitment of rictor to nucleus and the consequently activated mTORC2 phosphorylated the AKT at Ser473 (Zong et al., 2014; Prakash et al., 2019).

Primary cultured mouse tracheal epithelial cells (MTECs) originate from the proximal trachea and are composed of basal, goblet, club, ciliated, tuft, KRT13/4^+^, pulmonary neuroendocrine epithelial cells and ionocytes (Montoro et al., 2018). Due to the consistency with the morphology, physiology, transcriptional character, and cell types with tracheal epithelium *in vivo*, MTECs are widely applied in toxicity, viruses, and pharmacology-related experiments (Davidson et al., 2000; Horani et al., 2013; Hou et al., 2019; Ruiz Garcia et al., 2019; Carraro et al., 2021). In this study, we explore the mechanism of the hypoxia-induced EMT process by analyzing the single-cell sequencing data set of MTECs and culturing them in a liquid–liquid interface (LLI) mode to mimic the pathological status of respiratory diseases. Our data demonstrate that EMT occurs mainly in basal cells and is partly attributed to ribosome biogenesis under hypoxia, which provides a novel insight into hypoxia-related fibrosis of chronic respiratory diseases.

## Methods and Materials

### Cell Culture and Hypoxia Model

All experiments involving animals were performed in accordance with the guidelines and regulations of the Animal Care and Use Ethics Committee, China Medical University. The experimental protocols were approved by China Medical University, and the certificate number is SYXK (Liao) 2018-0008. Isolation and culture of MTECs have been described previously (Hou et al., 2019; Hou et al., 2020b). Briefly, we isolated the trachea from diazepam (17.5 mg kg^−1^ intraperitoneally) followed 6 min later by ketamine (450 mg kg^−1^ intraperitoneally) anesthetized mice and digested it with 0.1% protease ⅩⅣ and 0.01% DNase I (Sigma, St. Louis, MO, United States) in high-glucose DMEM containing 1% FBS (Gibco, New York, NY, United States) at 4°C on a horizontal rotator for 24 h. Cells were collected by the centrifugation of supernatant after the tracheal bones were sucked out and seeded at a density at 3.0 × 10^5^/cm^2^ on a collagen I (Gibco, New York, NY, United States) precoated transwell insert (3413, Corning-Costar, Lowell, MA, United States). The basolateral culture medium was changed every other day, and the apical culture medium was discarded to establish the air–liquid interface (ALI) culture mode after the transmembrane resistance was higher than 1000 Ω*cm^2^ measured by an epithelial voltohmmeter (WPI, Sarasota, FL, United States). After being cultured in this mode for another 13–15 days, MTECs in the LLI groups were treated by administration of 150 μl culture medium to the apical side for hypoxia cell model establishment. Ribosome biogenesis was inhibited by administration of 100 nM CX-5461 (Adooq, Irvine, CA, United States).

The H441 cell line was derived from human Clara cells found in the bronchiolar epithelium and grown in a six-well plate with 2 ml medium containing 10% FBS and 1% penicillin/streptomycin, whereas 8 ml medium or CoCl_2_ (300 μM), a chemical inducer of hypoxia by replacing the Fe^2+^ with Co^2+^ in the catalytic center of prolyl hydroxylase domain proteins (Muñoz-Sánchez and Chánez-Cárdenas, 2019), was administrated after the H441 cells reached 80% confluence. The H441 cell line was purchased from American type culture collection and passaged fewer than 30 times.

### Determination of Lactic Acid

The MTEC culture medium was collected on 0, 2, 4, and 8 days after the LLI culture, respectively. The lactate concentration was determined by a lactic content assay kit (Solarbio, Beijing, China), according to the manufacturer’s manual. Lactate production was normalized with the cell number.

### Morphology Studies

MTECs were fixed in 4% paraformaldehyde, dehydrated in 30% sucrose, embedded in optimal cutting temperature compound, and cut into 8 μm slices. HE staining was performed by the HE staining kit (Solarbio, Beijing, China) according to the manufacturer’s instructions.

For the immunofluorescence assay, Triton-100 (0.1%) was employed to permeabilize the cell membrane. To visualize the cells, we first incubated MTECs with Vimentin and Krt5 primary antibody at 4°C overnight and then with secondary antibodies for 90 min at room temperature. The nucleus was stained by DAPI, and all processes were done in a dark humidifying box.

### Western Blot Assay

Equivalent protein extracted from MTECs by RIPA was separated on SDS-page after the concentration was determined by a BCA kit (Solarbio, Beijing, China) and transblotted onto 0.45 μm PVDF membranes (Invitrogen, Waltham, MA, United States), which were blocked by 5% BSA for 1 h at room temperature and incubated with primary antibodies overnight at 4°C. Before and after the incubation with secondary antibodies, membranes were washed three times with TBST for 10-min intervals. The sources and dilution of all antibodies are listed in Supplementary Table S1. The images were developed by the ECL kit (Tanon, Shanghai, China) and analyzed with Image J software.

### Real-Time PCR Experiment

RNA was extracted by TRIzol reagent (Invitrogen, Waltham, MA, United States), and 500 ng RNA was used as a template for reverse transcription using the PrimeScript RT reagent Kit with gDNA Eraser (TaKaRa, Kusatsu, Shiga, Japan) after the concentration was determined by spectrophotometry at 260 nm. Reaction for all primers was performed using a single cycle of 95°C for 0.5 min, followed by 40 cycles of 95°C for 5 s, and 60°C for 34 s in the ABI 7500 real-time PCR System. Specific mRNA primers were validated by PrimerBank (https://pga.mgh.harvard.edu/primerbank/index.html), and to test the specificity of rRNA primers, products after real-time PCR were also loaded onto 1.2% agarose gel and visualized under ultraviolet light (Supplementary Figure S1). All primers are listed in Supplementary Table S2. Relative expression of RNA was calculated by 2^−ΔΔCT^, and *Actb* (β-actin) was used as an internal reference.

### Bioinformatics Analysis

The MTEC data set (GSE103354) consisted of airway epithelial cells from six healthy mice and was downloaded from the Gene Expression Omnibus database. Suerat v3 was used to analyze single-cell sequencing data, which were filtered by number of detected genes (50 < ngenes < 3200) and mitochondrial percentage (mito. pc < 5%). The filtered gene–barcode matrix was first normalized using the “LogNormalize” methods in Seurat v.3 with default parameters, and the top 1500 variable genes were then identified using the “vst” method in Seurat FindVariableFeatures function. Principal component analysis (PCA) was performed using the top 20 principal components, and the resolution was set to 0.15 to obtain a finer result in FindCluster process. The cells were annotated by classical markers of different cell types and visualized by t-distributed stochastic neighbor embedding (t-SNE) (Supplementary Figure S2). Differentiation of gene expression levels in MTECs between basal and other cells were achieved by using Student’s *t*-test (FindAllMarkers function), whereas the min. pct was set to 0.01. KEGG was employed to analyze the enrichment of basal cell–specific expressed genes with default parameters. It was considered significant if Bonferroni adjusted *P* (*p*. adj) was less than .05.

### Flow Cytometry and Cell Sorting

MTECs were digested by trypsin-EDTA after being washed by PBS twice. Isolated MTECs were fixed with 4% paraformaldehyde for 15 min, permeabilized with Triton-100, blocked with 5% BSA in PBS before being incubated with primary and secondary antibodies, and the cell ratio was tested. For cell sorting, isolated MTECs were fixed and permeabilized with methanol, blocked with 5% BSA in PBS, incubated with Krt5, and sorted in flow cytometry (BD Arial Ⅱ). Antibodies are also listed in Supplementary Table S1.

### Statistical Analysis

Data are presented as the mean ± SE. We evaluated the power of the sample size first to meet *p* < .05. The differences between groups were tested by Student’s *t*-test or one-way analysis of variance (ANOVA) followed by Bonferroni’s test for all the groups of the experiment after the data passed the normality (Shapiro-Wilk) and homoscedasticity (Levene) tests. If the data did not pass the normality and homoscedasticity tests, a nonparametric *t-*test (Mann–Whitney U-test) was used to compare the differences between groups. Statistical analysis was performed with Origin 8.0.

## Results

### Hypoxia Model Establishment

**FIGURE 1:**
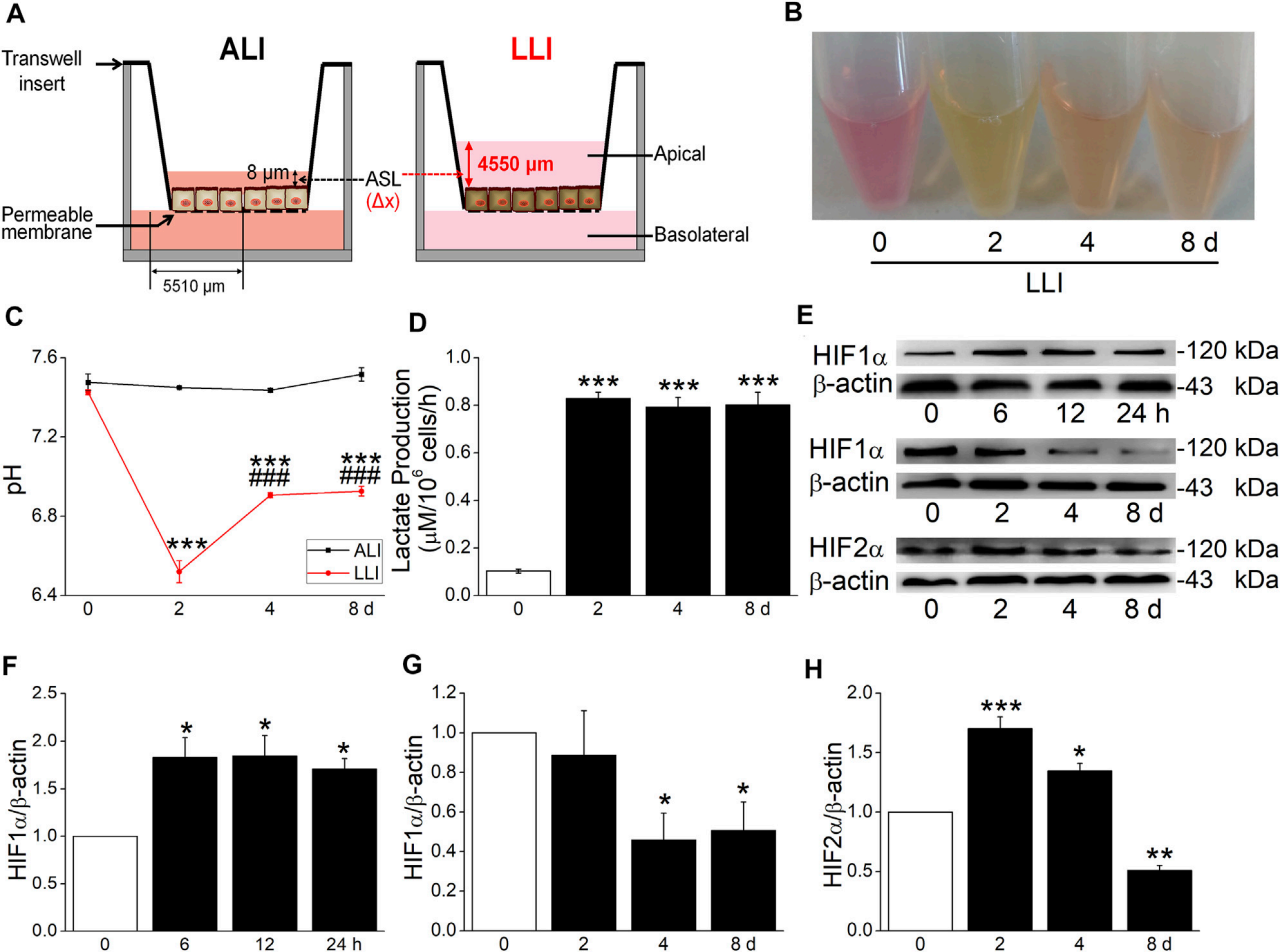
Hypoxia model of MTECs established. **(A)** A schematic diagram highlighting the distance of oxygen diffusion (Δ*x*) was different in ALI and LLI mode. The thickness of airway surface liquid was about 8 μm in ALI mode and 4550 μm in LLI mode. **(B–D)** Color, pH, and lactate production of MTEC medium after 0, 2, 4, and 8 days’ LLI culture. ^***^ *p* < .001, compared with the 0 day group, *n* = 3–6. One-way ANOVA followed by Bonferroni’s test was used to analyze the difference of the means for significance. **(E)** Representative graph of hypoxia-inducible factors (HIFs) after different time intervals in LLI mode. **(F–H)** The statistical data of HIF1α and HIF 2α in the mode of LLI culture. ^*^ *p* < .05, ^**^ *p* < .01, ^***^ *p* < .001, compared with ALI mode (0 h/d), *n* = 3–4. Mann–Whitney U test was used to analyze the difference of the means for significance in **(F)** and **(G)**, One-way ANOVA followed by Bonferroni’s test was used to analyze the difference of the means for significance in **(H)**.

To protect the airway from pathogens, the thin surface liquid layer lining on the mammalian epithelium of the airway is necessary for cilium beating and contaminant clearance, which also exists in MTECs. Due to various pathological causes in chronic respiratory diseases, the fluid retention in the airway/MTECs may result in epithelium hypoxia (Place et al., 2017; Hou et al., 2020a). In this experiment, we established a hypoxia model *in vitro* to mimic the abovementioned pathophysiology status. According to Fick’s first law, the velocity of the oxygen supplement was reduced by about 300 times after the culture mode of MTECs was changed from ALI to LLI (Figure 1A) and equal to that in a hypoxia incubator at 0.07% oxygen concentration, just between 0% and 1% oxygen concentration that was often employed in *in vitro* hypoxia-related respiratory studies (Planès et al., 1997; Polosukhin et al., 2011; Gerovac et al., 2014).

To further identify the hypoxia of MTECs, we observed the physical characteristics and lactate production of culture medium. As shown in Figure 1B, the color of medium in LLI mode was yellow, which showed pink in ALI mode, consistent with the pH manifestation in both conditions (Figure 1C). To quantify the difference in physical characteristics, the lactate production was measured, which showed higher in LLI groups (2, 4, and 8 days) than that in the ALI group (0 days, Figure 1D). As the indicator of hypoxia, HIF1α significantly increased within several hours after the MTEC culture mode was changed from ALI to LLI, whereas it decreased after being cultured for another several days. As for HIF2α, a prolonged increase happened on 2 and 4 days of LLI culture (Figures 1E–H). Meanwhile, excessive culture medium for submerged cells may be another theoretical hypoxia model *in vitro* according to Fick’s first law. As expected, HIF1α significantly increased in H441 cells submerged with 8 ml (6 ml redundant) of medium in a six-well plate with CoCl_2_ (300 μM, a chemical inducer of HIF1α) as the positive control (Supplementary Figure S3).

### Hypoxia Induces EMT in MTECs

**FIGURE 2:**
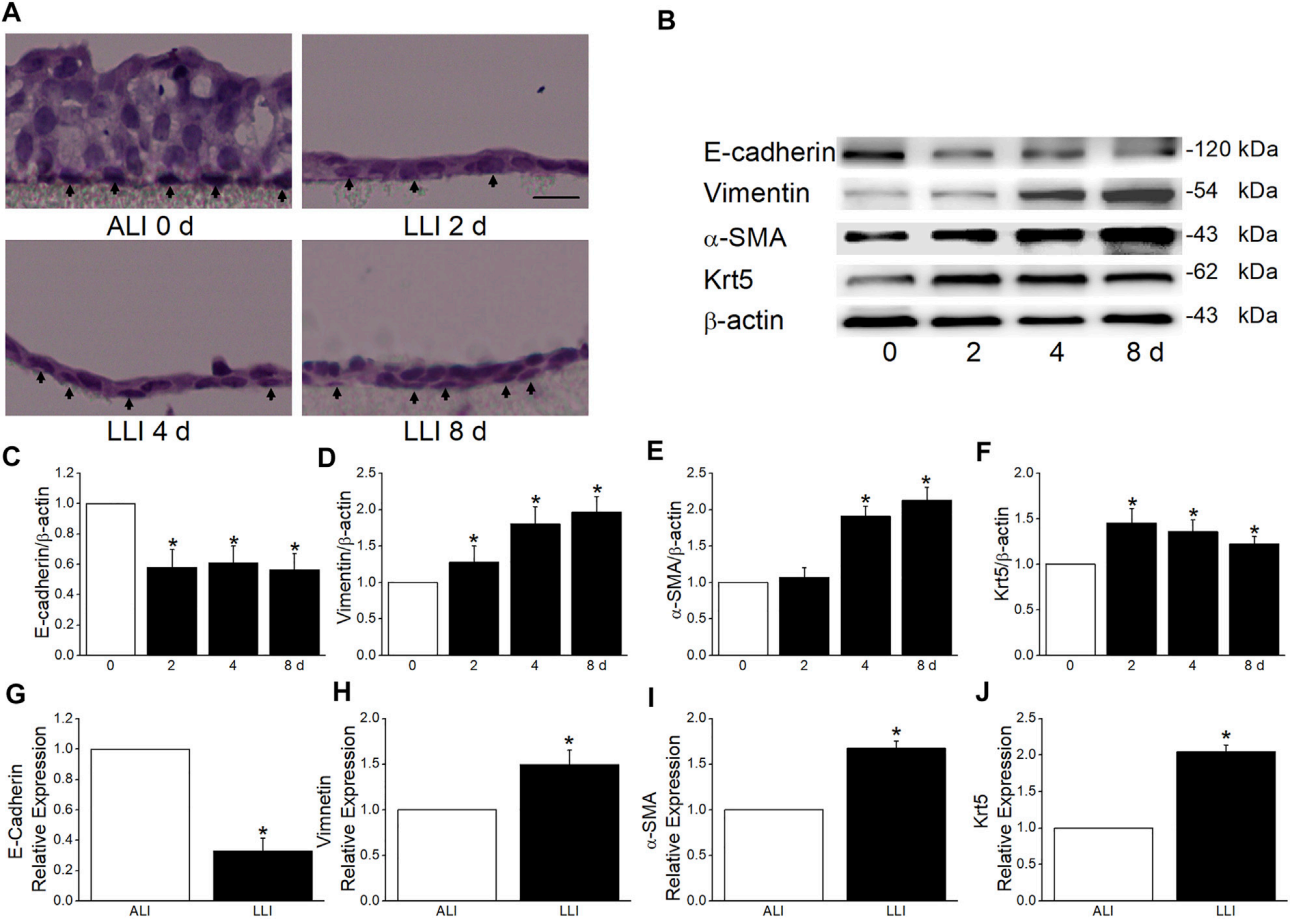
MTECs underwent EMT in the mode of LLI culture. **(A)** HE staining of MTECs after 0, 2, 4, and 8 days’ (d) LLI culture with the arrowheads indicating the representative cells in each panel. Scale bar = 10 μm. **(B)** Representative graph of EMT markers (E-cadherin, Vimentin, and α-SMA) and Krt5 after 0, 2, 4, and 8 days’ LLI culture. **(C–F)** The statistical data of EMT markers and Krt5 in the mode of LLI culture. ^*^ *p* < .05, compared with ALI group (0 days), *n* = 4. **(G–J)** Real-time PCR results for EMT marker and Krt5 mRNAs, and β-actin was used as internal reference. ^*^ *p* < .05, compared with ALI (0 days), *n* = 4–5. Mann–Whitney U test was used to analyze the difference of the means for significance.

To confirm the hypoxia induces EMT in respiratory epithelial cells, we applied a morphology assay and found that the type of MTECs close to the basolateral side was converted from cubic in ALI mode to spindle after 4 days’ LLI culture (Figure 2A). Additionally, the decreased/increased protein expression levels of epithelial/mesenchymal cell markers indicated the occurrence of EMT under hypoxia, which were also supported by the data from transcription level (Figures 2B–E,2G–I).

### Basal Cells Contribute to EMT Under Hypoxia

**FIGURE 3:**
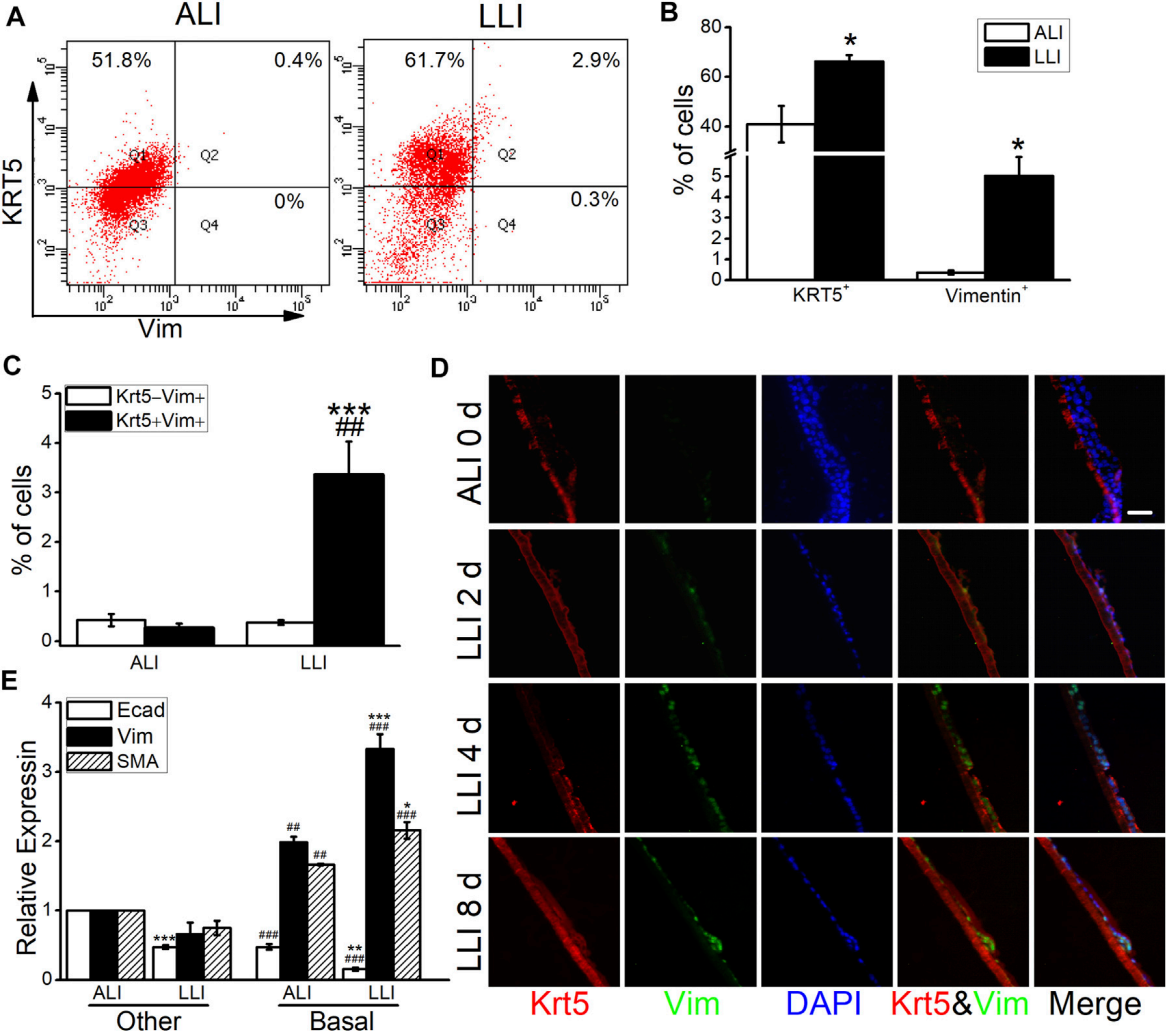
EMT induced by hypoxia was mediated by basal cells. **(A)** Representative data of flow cytometry in the ALI and LLI groups and the cell ratios were labeled in the corresponding gates. **(B)** Vimentin and Krt5 positive cell ratios in the mode of LLI culture. ^*^ *p* < .05, compared with ALI group, *n* = 3–4. **(C)** Krt5 positive cell ratio in Vimentin expressed cells. ^***^ *p* < .001, compared with Krt5^+^Vimentin^−^ cells in LLI group, ^##^ *p* < .01 compared with KRT5^+^Vimentin^+^ cells in ALI group, *n* = 3–5. **(D)** Co-expression of Krt5 (red) and Vimentin (green) in MTECs after LLI culture. Scale bar = 20 μm. **(E)** Heterogeneously expressed EMT markers in different airway epithelial cell types after LLI culture. ^*^ *p* < .05, ^**^ *p* < .01, ^***^ *p* < .001, compared with the same cell type, ^##^ *p* < .01, ^###^ *p* < .001, compared with the other cells in ALI/LLI group. *n* = 3. One-way ANOVA followed by Bonferroni’s test was used to analyze the difference of the means for significance.

The hyperplasia of basal cells after hypoxia was observed in our study (Figures 2B,F,J), implying the involvement of basal cells in hypoxia-induced EMT. Consequently, our flow cytometry result identified that the Krt5 and Vimentin (basal and mesenchymal marker, respectively) positive cells both increased in LLI mode, and most of the Vimentin positive cells also expressed Krt5 (Figures 3A–C), indicating that the mesenchymal cells may be mainly originated from basal cells. The direct image from the immunofluorescence assay showed that the Vimentin-positive cells obviously increased after 4 days’ LLI culture, which were mostly Krt5 positive (Figure 3D). To affirm the role of basal cells in hypoxia-induced EMT, we separated basal from other cells in MTECs by flow cytometry, and as expected, the E-cadherin (epithelial cell marker) mRNA decreased while the Vimentin and α-SMA (mesenchymal cell markers) mRNA increased more significantly in basal cells compared with the other cells in the LLI group (Figure 3E, *p* < .001, *n* = 3), indicating that basal cells in MTECs contribute to EMT occurrence under hypoxia.

### Ribosome Genes are Highly Expressed in Basal Cells

**FIGURE 4:**
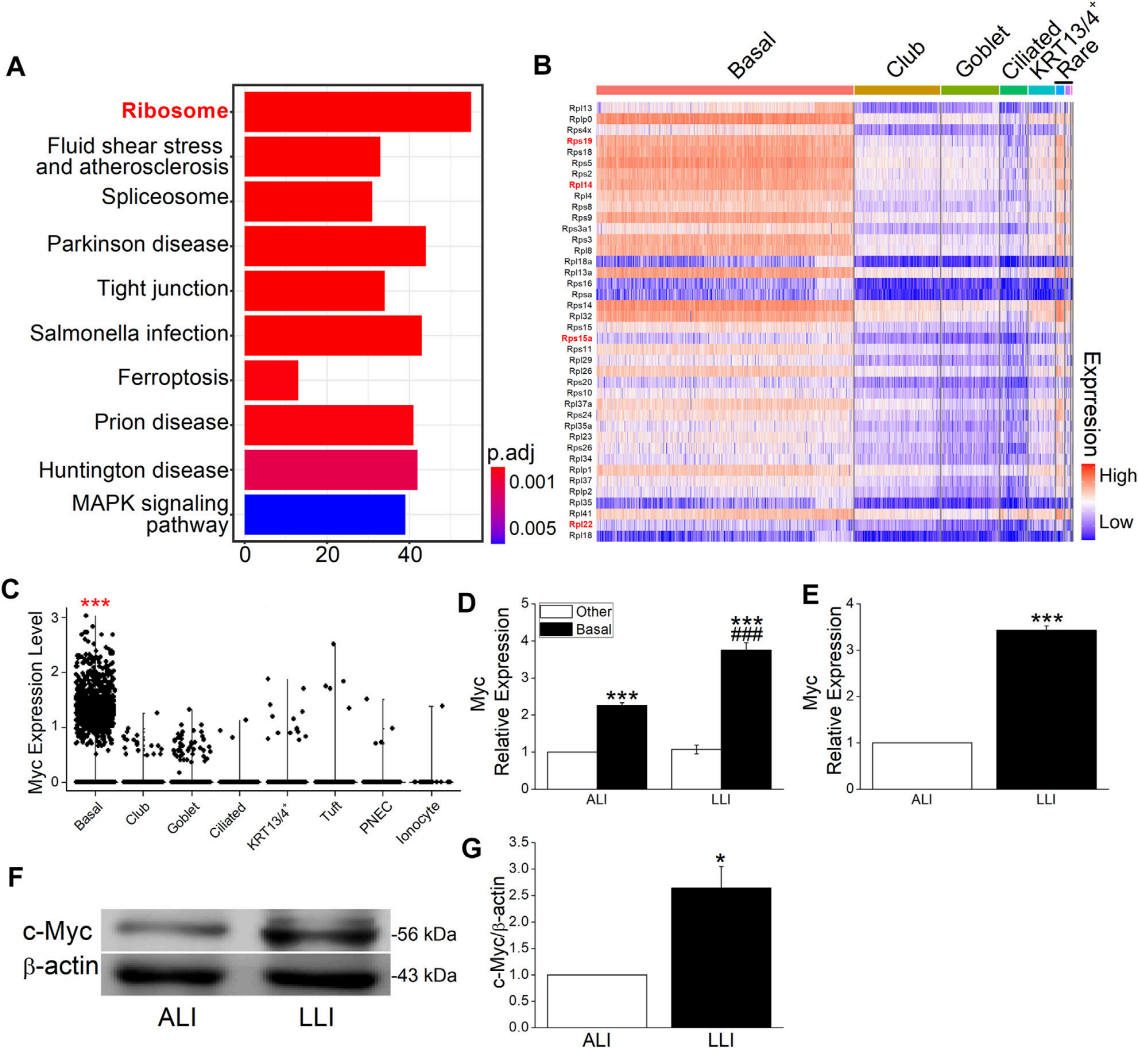
Myc and ribosome proteins were enriched in basal cells. **(A)** Top 10 enriched pathways of specifically expressed genes in basal cells. Numbers in *x*-axis represent the genes enriched in the corresponding pathway. *P* adjust (*p*. adj) varies from .01 to 0.05. **(B)** Top 40 ribosome proteins among different cell types. The red and bold font marked genes were closely associated with EMT. **(C)** Myc in different airway epithelial cells. ^***^ *p*. adj <.001. **(D)** Heterogeneously expressed Myc mRNA in different airway epithelial cell types after LLI culture. ^***^ *p* < .001 compared with the other cells in corresponding group. ^###^ *p* < .001, compared with ALI group within the same cell type. *n* = 3. One-way ANOVA followed by Bonferroni’s test was used to analyze the difference of the means for significance. **(E)** Expression of Myc mRNA in ALI and LLI group. ^***^ *p* < .001, compared with ALI group, *n* = 3. Student’s *t*-test was used to analyze the difference of the means. **(F–G)** Representative and statistical data of c-Myc. ^**^ *p* < .01, compared with ALI group, *n* = 4. Mann–Whitney U test was used to analyze the difference of the means for significance.

More and more evidence proves the basal cell as the main cell type that contributes to EMT in different tissues, but the reason was still unclear (Liu et al., 2016; Xu et al., 2016; Zhang et al., 2016; Xu et al., 2017). To find the possible explanation of basal cell but not other cell types in hypoxia-induced EMT, we analyzed the single-cell sequencing data set of MTECs and found that the differently expressed genes between basal and other cells were enriched in ribosome, fluid shear stress and atherosclerosis, spliceosome, and so on (Figure 4A).

The most enriched ribosome was reported to be closely associated with EMT in breast cancer (Prakash et al., 2019; Dermit et al., 2020), whereas its role in hypoxia-related respiratory diseases still needs further exploration. The heat map shows the top 40 ribosome protein genes ranked by the decreased fold change of expression, and the red marks represent the known EMT-related genes in previous publications (Figure 4B). As the global regulator of ribosome biogenesis, Myc was significantly highly expressed in basal cells as shown in the violin plot (Figure 4C, p. adj <.001) and proved to be enriched in separated basal cells (Figure 4D, *p* < .001, *versus* the other cells, *n* = 3). Whereas in the mode of LLI culture, the expression of Myc increased significantly in basal cells compared with that in ALI mode (Figure 4D, *p* < .01, *n* = 3). Identical with the result of separated cells, Myc expression in bulk MTECs were significantly increased in LLI mode at mRNA and protein levels (Figures 4E–G, *p* < .001–.05, *versus* ALI, *n* = 4), supporting that ribosome may be involved in hypoxia-induced EMT in basal cells.

### Ribosome Biogenesis is Increased Mainly in Basal Cells Under Hypoxia

**FIGURE 5:**
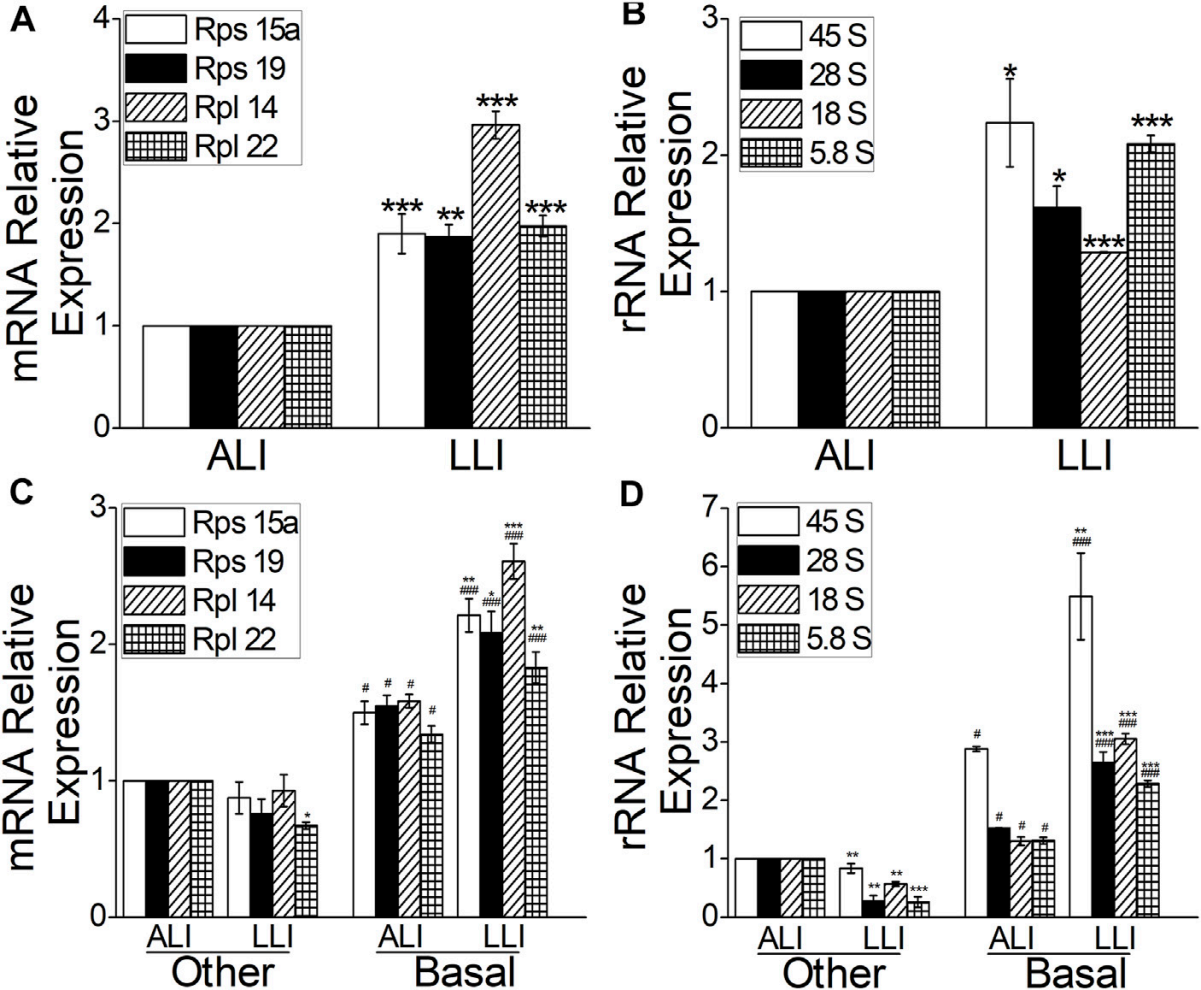
Ribosome biogenesis was enhanced in the mode of the LLI culture. **(A–B)** Real-time PCR results of ribosome biogenesis–related RNAs (Rps15a, Rps19, Rpl14, and Rpl22 as well as 45, 28, 18, and 5.8S rRNA). ^*^ *p* < .05, ^**^ *p* < .01, ^***^ *p* < .001, compared with ALI group, *n* = 3. Student’s *t*-test was used to analyze the difference of the means. **(C–D)** Ribosome biogenesis–related RNAs were significantly upregulated in basal cells in the mode of LLI culture. ^*^ *p* < .05, ^**^ *p* < .01, ^***^ *p* < .001 compared with ALI group within same cell type, ^#^ *p* < .05, ^###^ *p* < .001, compared with the other cells in corresponding group. *n* = 3. One-way ANOVA followed by Bonferroni’s test was used to analyze the difference of the means for significance.

To test whether ribosome increased in airway hypoxia, we measured the expression of ribosome-related RNAs (including rRNA and mRNA) in bulk and separated MTECs, respectively. Both mRNA and rRNA increased significantly in LLI mode (Figures 5A,B, *p* < .001–.05, *versus* ALI, *n* = 4), indicating that ribosome biogenesis increased under hypoxia in airway epithelium. The data were also proved in separated basal cells (Figures 5C,D, *p* < .001, *versus* the other cells, *n* = 3) cultured in LLI mode, which implies the aforementioned hypoxia increased ribosome biogenesis mainly occurred in basal cells.

### Inhibition of Ribosome Biogenesis Abrogates the EMT Process

**FIGURE 6:**
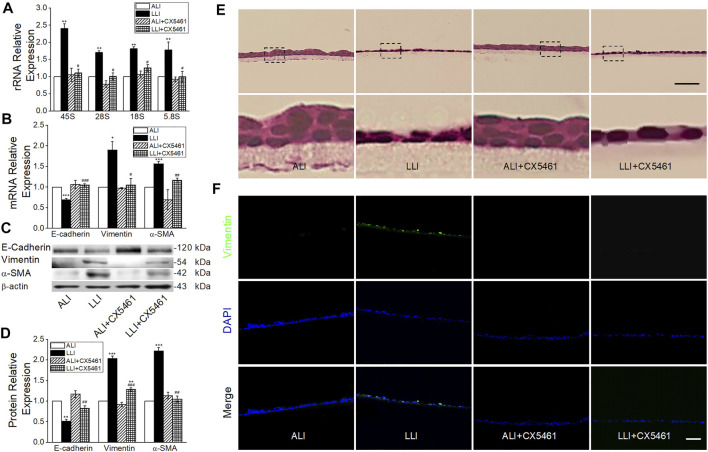
CX-5461 abrogated hypoxia-induced EMT. **(A)** rRNA expression level after administration of 100 nM CX-5461 in the mode of LLI culture. ^**^ *p* < .01, compared with ALI group, ^#^ *p* < .05 compared with LLI group, *n* = 5. Mann–Whitney U test was used to analyze the difference of the means for significance. **(B)** Expression of E-cadherin, Vimentin, and α-SMA. ^*^ *p* < .05, ^***^ *p* < .001, compared with ALI group, ^#^ *p* < .05, ^##^ *p* < .01, ^###^ *p* < .001, compared with LLI group, *n* = 3. **(C–D)** Representative and statistical data of EMT markers. ^**^ *p* < .01, ^***^ *p* < .001 compared with ALI group, ^##^ *p* < .01, ^###^ *p* < .001, compared with LLI group, *n* = 3–4. One-way ANOVA followed by Bonferroni’s test was used to analyze the difference of the means for significance. **(E)** HE staining. The nether line of images was enlarged from the upper original image. **(F)** Immunofluorescence of Vimentin (green). The nucleus was stained with DAPI (blue). Scale bar = 50 μm.

Ribosome biogenesis was increased under hypoxia, but whether it facilitated the EMT process was still unknown. CX-5461, a specific RNA polymerase Ⅰ inhibitor that blocks ribosome biogenesis, significant decreased the transcription of rRNAs in LLI mode (Figure 6A, *p* < .05, *n* = 5) and almost offset the corresponding change of EMT markers at both the mRNA and protein levels in LLI mode (Figures 6B–D, *p* < .001–.05, *n* = 3–4). Consistently, the HE staining result showed that CX-5461 mitigated the morphology changes of MTECs, and most of them were still cubic in the mode of LLI culture (Figure 6E). In Figure 6F, Vimentin-positive cells increased in LLI mode, and were significantly reduced by administration of CX-5461. Based on the above, we suppose that the occurrence of EMT under hypoxia in basal cells was mainly mediated by increased ribosome biogenesis.

### mTORC2 and AKT are Involved in Hypoxia Induced EMT in MTECs

**FIGURE 7:**
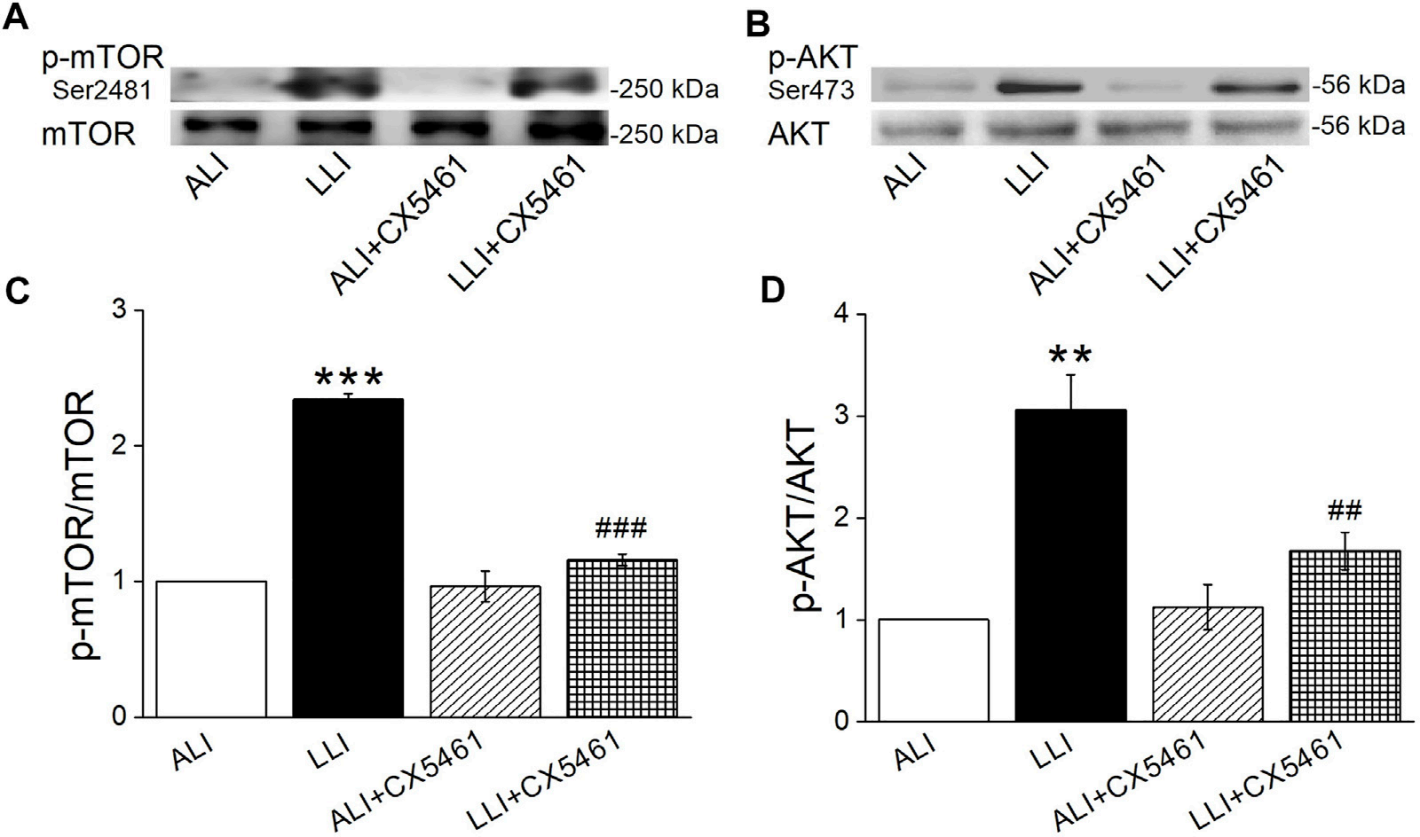
Effect of CX-5461 downstream of ribosome. **(A, C)** Representative and statistical data of mTOR phosphorylation at Ser2481 after CX-5461 treatment. **(B, D)** Representative and statistical data of AKT phosphorylation at S473 after CX-5461 treatment. ^***^ *p* < .001 compared with ALI group, ^###^ *p* < .001, compared with LLI group, *n* = 3–4. One-way ANOVA followed by Bonferroni’s test was used to analyze the difference of the means for significance.

**FIGURE 8:**
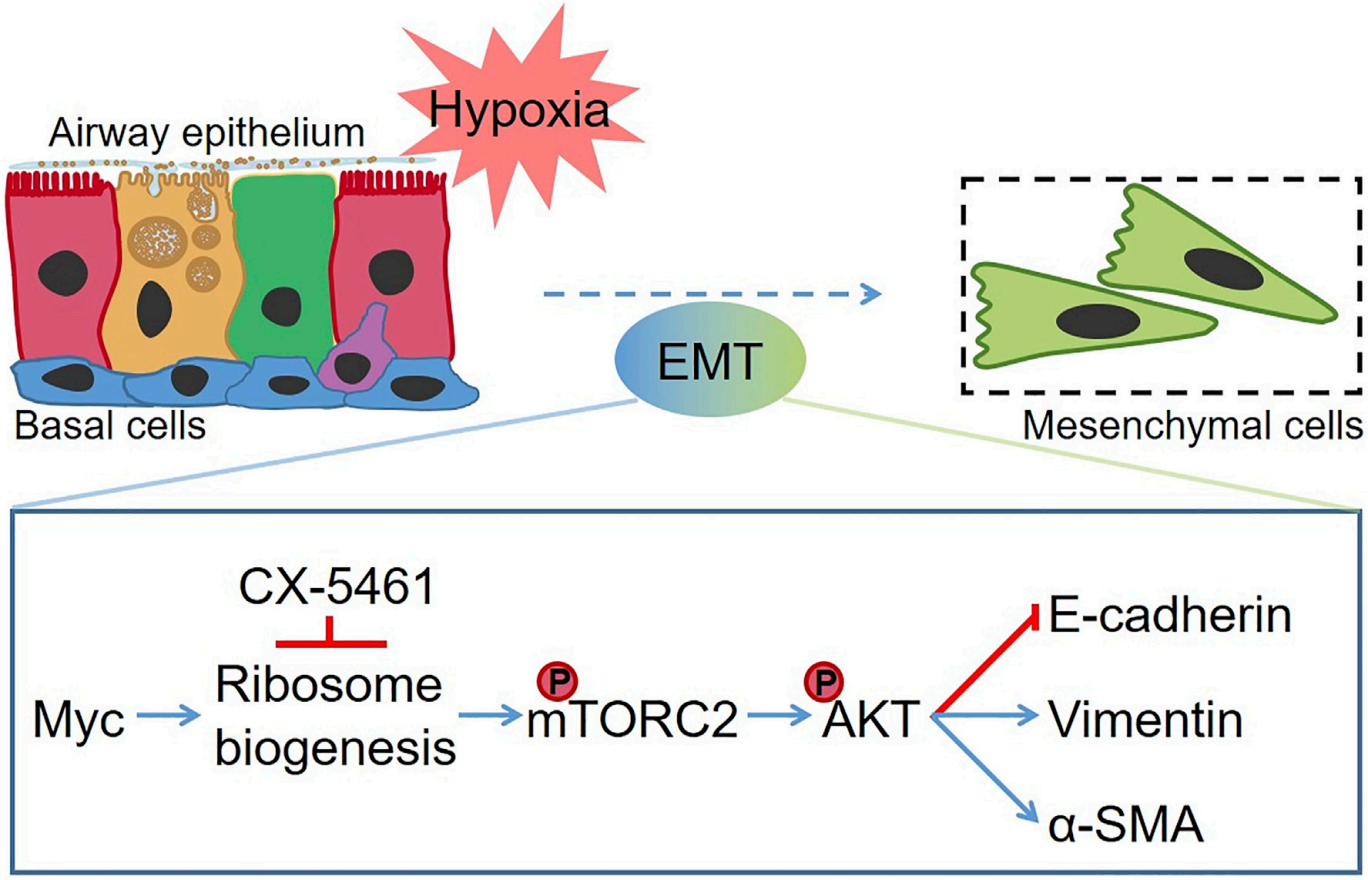
The schematic diagram of hypoxia-induced EMT in MTECs. Hypoxia enhanced the ribosome biogenesis by increasing the expression of Myc. Subsequently, the phosphorylation of mTOR at Ser2481 was upregulated, and phosphorylation of AKT significantly increased, which promotes the occurrence of the EMT process. CX-5461 suppressed hypoxia-induced EMT by inhibiting the ribosome biogenesis. The red line denotes the repression effects, and the blue line indicates the facilitation effect.

To explore the mechanism involved in ribosome-mediated EMT, we measured the phosphorylation of mTOR (Ser2481), which was a component of EMT closely associated mTORC2 and activated by the binding of ribosome (Copp et al., 2009; Karimi Roshan et al., 2019). As shown in Figures 7A,C, CX-5461 significantly alleviated the mTOR phosphorylation in LLI mode (*p* < .001, *n* = 4). Similarly, the phosphorylation of AKT at Ser473, a downstream effector of mTORC2, was significantly increased in LLI mode, which could be suppressed by CX-5461 (Figures 7B,D, *p* < .01, *n* = 3). These data indicate that hypoxia-induced EMT in basal cells was mainly mediated by ribosome-mTORC2-AKT axis (Figure 8).

## Discussion

In this study, we first established a hypoxia model by culturing MTECs in LLI mode, which mimicked the retention of fluid in the airway under pathological conditions of many chronic respiratory diseases. Hypoxia was verified with the color, pH, and lactic concentration of culture medium as well as HIF1α and HIF2α expression in MTECs. Compared with the hypoxic incubator, which decreases the concentration gradient of the oxygen on either side of the medium (ΔC in Fick’s first law), LLI culture mode increases the thickness (Δx) of the medium, both of which belong to the physical method to induce hypoxia. Consistent with previous studies that HIF1 and HIF2 governed acute and prolonged hypoxia in the human endothelium, respectively, we found that HIF1α was upregulated within several hours, whereas it was decreased in a prolonged airway epithelium hypoxia. Meanwhile, the expression of HIF2 increased after 4 days’ LLI culture, reflecting the switch from HIF1 to HIF2 signaling in adapting the epithelium to prolonged hypoxia (Torres-Capelli et al., 2016; Serocki et al., 2018).

Hypoxia can influence the composition of cell types, tight junctions, and ion channels in respiratory diseases (Jimenez et al., 2016; Torres-Capelli et al., 2016; Bartoszewska et al., 2017). Hypoxia can promote the differentiation of basal cells to goblet cells, and excessive mucus secreted by the latter aggravates airway hypoxia and affects the differentiation process of basal cells (Polosukhin et al., 2011). A previous study shows that hypoxia-induced airway fibrosis mainly occurs in proximal, medium tracheal, whereas the distal trachea may have a synergistic effect on matrix collagen degradation (Broytman et al., 2015). Although the generations and spatial heterogeneity of cells in the airway are different between mice and humans, the cell types, differentiation characteristics, and morphology in the airway epithelium of mice are basically identical with that of human, and studies of the respiratory fibrosis in mice remain the most clinically relevant model for the preclinical study of human respiratory fibrosis at present. Accordingly, MTECs may be a selectable appropriate cell model to study the mechanisms of hypoxia-induced EMT (Hogan et al., 2014; Tashiro et al., 2017; Basil and Morrisey, 2020).

Here, we report that hypoxia induces EMT in MTECs, which play a key role in cystic fibrosis, asthma, and other airway diseases. Meanwhile, we found the ratio of basal cells increased under the hypoxia condition, and these were regarded as the progenitor cells for the other airway epithelial cells and the culprit of EMT in human fibrotic diseases. However, the effect of hypoxia on ionocytes, tuft, KRT13/4^+^, and pulmonary neuroendocrine epithelial cells still needs further study (Kuroishi et al., 2009; Xu et al., 2016; Bankova et al., 2018; Montoro et al., 2018; Sui et al., 2018).

To explore the possible mechanisms involved in basal cells participating in EMT under hypoxia, we analyzed the single-cell sequencing data set and found that basal cell–specific genes were enriched in ribosome, which has been recently reported to be related to EMT. As the components of ribosome, Rps15a, Rps19, Rpl14, Rpl22 were preciously controlled by Myc and highly expressed in cancer cells, which promoted the EMT process (Chen et al., 2018; Wang et al., 2018; Feng et al., 2019; Liu et al., 2019). Accordingly, besides its acting as the protein synthesis machine, the role of ribosome in signal transduction needs to be considered. Ribosome binds and activates mTORC2 at least by Rpl26, Rps16, which were also included in the top 40 enriched ribosome genes of basal cells, according to our single-cell analysis (Zinzalla et al., 2011).

AKT downstream of mTORC2, can be activated during the process of EMT. In our experiment, inhibition of ribosome biogenesis suppressed the phosphorylation of both mTOR and AKT, which may influence the protein expression level of EMT transcription factors, matrix metalloproteinases, and numerous EMT-related pathways (Gulhati et al., 2011; Karimi Roshan et al., 2019; Fu and Hall, 2020). Our main finding in this study is that basal cells participate in hypoxia-induced EMT mainly by the ribosome-mTORC2-AKT axis, and there may be other reasons, such as the enriched MAPK pathway, as well as highly expressed genes including *Snai2*, *Id1*, *Sparc*, *Tgfb1*, and *Rhoa* visualized by the nferX single-cell sequencing portal (https://academia.nferx.com) in basal cells (Song et al., 2018; Ma et al., 2019; Sangaletti et al., 2019; Zhao et al., 2020). We believe that our findings would provide a new idea for future prevention and treatment in hypoxia related fibrosis.

## Conclusion

Hypoxia-induced EMT was mainly contributed by basal cells *via* ribosome-mTORC2-AKT axis in MTECs.

## Data Availability

The original contributions presented in the study are included in the article/Supplementary Material, and further inquiries can be directed to the corresponding authors.

## Abbreviations

ALI, air-liquid interface; EMT, epithelial-mesenchymal transition; HIF, Hypoxia-inducible factors; LLI, liquid-liquid interface; MTECs, mouse tracheal epithelial cells; mTORC2, mammalian target of rapamycin complex 2.
