# Supplementary material for: Airway Basal Cells Mediate Hypoxia-Induced EMT by Increasing Ribosome Biogenesis
Source: Front Pharmacol. 2021 Dec 9;12:783946. doi: 10.3389/fphar.2021.783946 (PMC8696177; doi:10.3389/fphar.2021.783946)
Supplement: Supplementary file 1 [file DataSheet1.ZIP › Supplementary materials/Supplementary file.docx]

**Supplementary information**

**Airway basal cells mediate hypoxia induced EMT by increasing ribosome biogenesis**

Yapeng Hou ^1^, Yan Ding ^1^, Danni Du ^2^, Tong Yu ^1^, Wei Zhou ^1^, Yong Cui ^2,^ *, Hongguang Nie ^1,^ *

^1^ Department of Stem Cells and Regenerative Medicine, College of Basic Medical Science, China Medical University, Shenyang, China

^2^ Departments of Anesthesiology, the First Hospital of China Medical University, Shenyang, China

* Corresponding authors:

Hongguang Nie: [hgnie@cmu.edu.cn](mailto:hgnie@cmu.edu.cn), Yong Cui: ycui@cmu.edu.cn

**Supplementary Table 1.**

The relative information of antibodies

| Antibody | Manufacture | Catalog Num. | WB Dilution | IF Dilution | FCM/FACS Dilution |
| --- | --- | --- | --- | --- | --- |
| E-Cadherin | Affinity | AF0131 | 1:1000 |  |  |
| Vimentin | Proteintech | 10366-1-AP | 1:1000 | 1:100 |  |
| α-SMA | Merck | CBL171 | 1:1000 |  |  |
| Krt5 | Elabsciecnce | E-AB-31148 | 1:1000 | 1:100 |  |
| Krt5 | HUABIO | EM1706-36 |  |  | 1:1000 |
| Hif 1α | Affinity | AF1009 | 1:1000 |  |  |
| Hif2α | Santa Cruz | sc-13596 | 1:1000 |  |  |
| c-Myc | Abmart | T55150F | 1:1000 |  |  |
| Phospho-mTOR | Affinity | AF3309 | 1:500 |  |  |
| mTOR | Affinity | AF6308 | 1:1000 |  |  |
| Phosphor-AKT | Affinity | AF0016 | 1:1000 |  |  |
| AKT | Affinity | AF6261 | 1:1000 |  |  |
| β-actin | Santa Cruz | sc-47778 | 1:1000 |  |  |
| Goat-anti-mouse | ZSGB-bio | ZB-2305 | 1:5000 |  |  |
| Goat-anti-rabbit | ZSGB-bio | ZB-2301 | 1:5000 |  |  |
| Rhodamine-goat-anti-mouse | ZSGB-bio | zf-0313 |  | 1:200 |  |
| FITC-goat-anti-rabbit | ZSGB-bio | ZF-0311 |  | 1:200 | 1:200 |
| AF647-goat-anti-mouse | Immunoway | RS3808 |  |  | 1:5000 |

IF: immunofluorescence, FCM: flow cytometry, FACS: fluorescence activated cell sorting, WB: western blot.

**Supplementary Table 2.**

The primers for real-time PCR

| Protein/rRNA name | Gene name | Forward (5'-3') | Reverse (5'-3') |
| --- | --- | --- | --- |
| Vimentin | Vim | CGTCCACACGCACCTACAG | GGGGGATGAGGAATAGAGGCT |
| α-SMA | Acta2 | GTCCCAGACATCAGGGAGTAA | TCGGATACTTCAGCGTCAGGA |
| E-Cadherin | Cdh1 | CAGGTCTCCTCATGGCTTTGC | CTTCCGAAAAGAAGGCTGTCC |
| KRT5 | Krt5 | TCTGCCATCACCCCATCTGT | CCTCCGCCAGAACTGTAGGA |
| Rps19 | Rps19 | CAGCAGGAGTTCGTCAGAGC | CACCCATTCGGGGACTTTCA |
| Rpl14 | Rpl14 | GGGTGGCCTACATTTCCTTCG | CTTGGCCCATCTTGTGGCT |
| Rps15a | Rps15a | GCATCAACAACGCTGAGAAGA | ACCAATGTATCCGTGCTTCATC |
| Rpl22 | Rpl22 | AGCAGGTTTTGAAGTTCACCC | CAGCTTTCCCATTCACCTTGA |
| 45S rRNA | Rn45s | ACACGCTGTCCTTTCCCTATTA | CCCAAGCCAGTAAAAAGAATAGG |
| 28S rRNA | Rn28s1 | TACGAATACAGACCGTGAAAGC | CTGTGGTAACTTTTCTGACACC |
| 18S rRNA | Rn18s | CTATTTTGTTGGTTTTCGGAACTG | TAATGAAAACATTCTTGGCAAATGCT |
| 5.8S rRNA | Rs5-8s1 | CTCTTAGCGGTGGATCACTC | GAAGTGTCGATGATCAATGTGTC |
| Myc | Myc | ATGCCCCTCAACGTGAACTTC | CGCAACATAGGATGGAGAGCA |
| β-actin | Actb | GGCTGTATTCCCCTCCATCG | CCAGTTGGTAACAATGCCATGT |


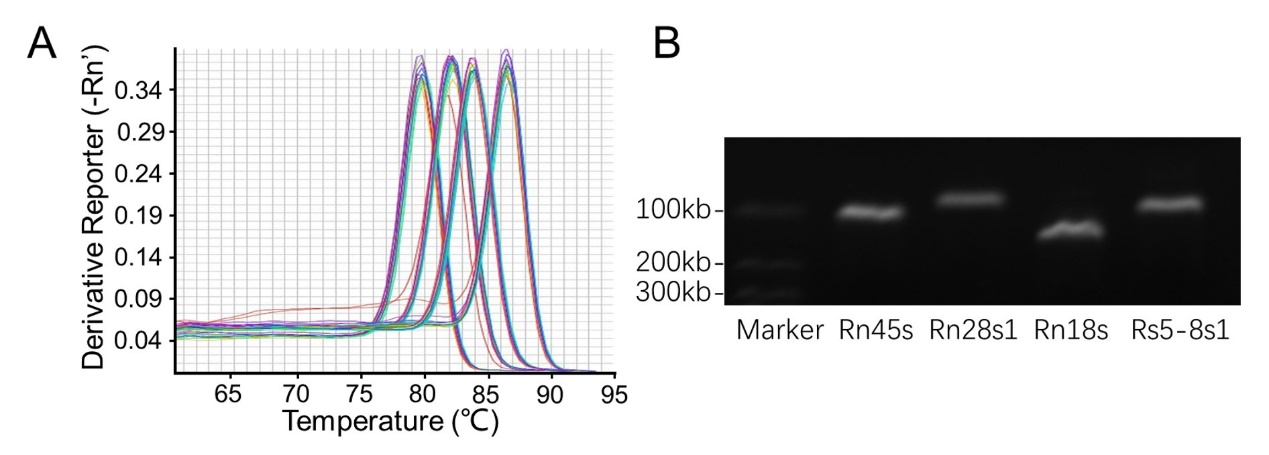


**Supplementary Figure 1.** Specification of rRNA primers. (A) Characteristic melt peak of 45S, 28S, 5.8S, and 18S rRNA appeared at 79.5℃, 82℃, 84℃, and 86.5℃, respectively. (B) Agarose gel electrophoresis showed 102, 86, 140, and 99 bp products amplified using 45S, 28S, 18S, and 5.8S rRNA specific primers, respectively.


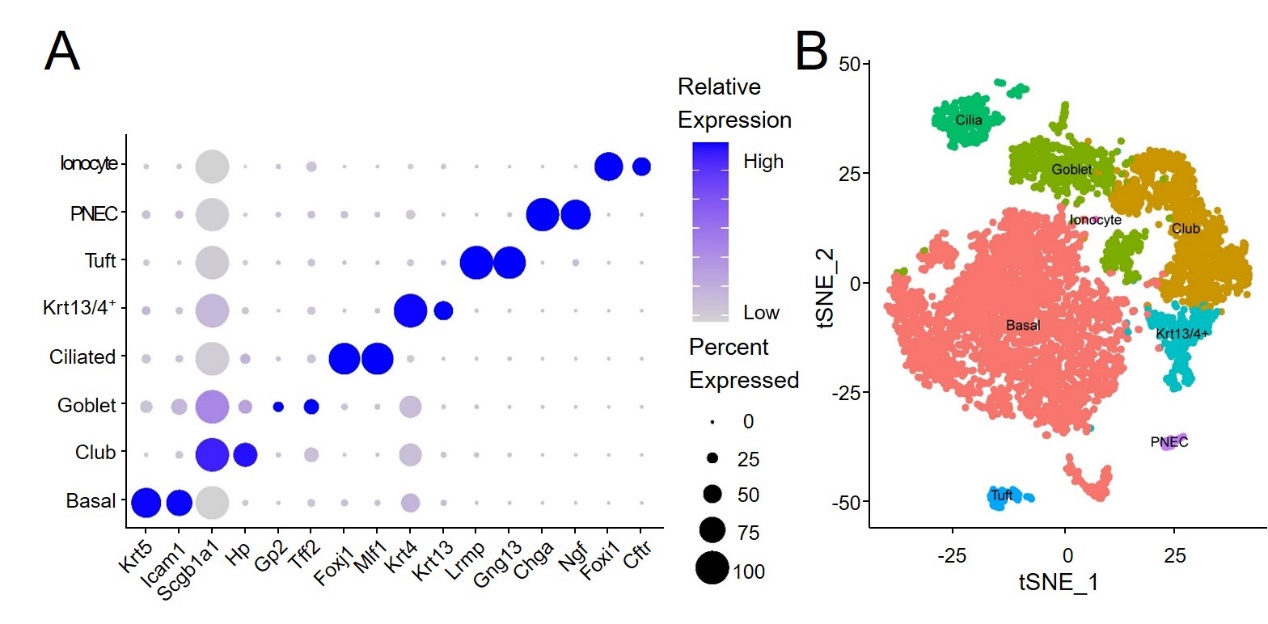


**Supplementary Figure 2.** Markers used for cell annotation in single-cell sequencing data analysis. (A) Bubble plot showed the markers used for MTECs annotation. The size of the dot represented the cell ratio, while the color represented the gene expression level. (B) t-SNE plot of MTECs after cell annotation.


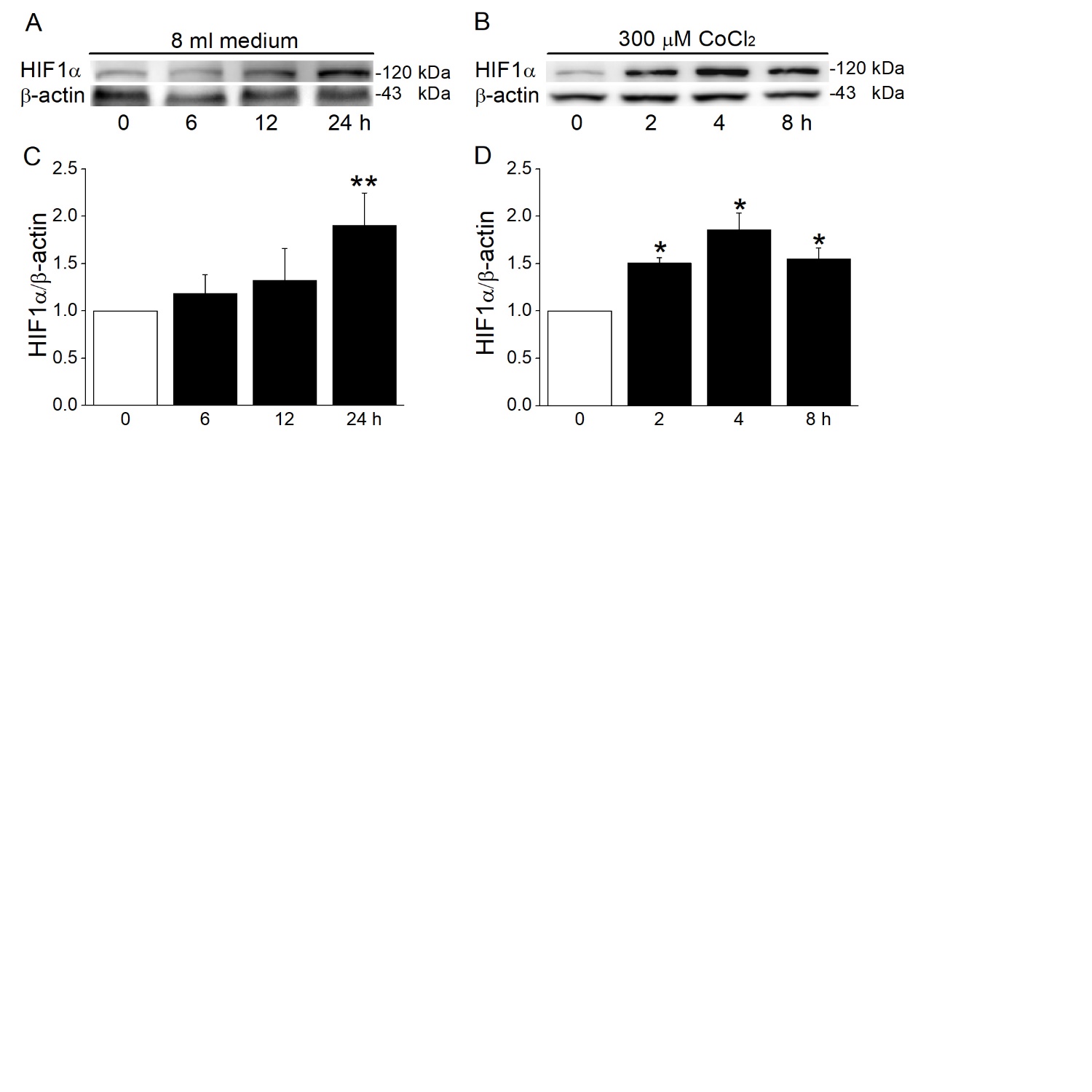


**Supplementary Figure 3.** Excessive medium induced hypoxia in H441 cells. (A, C) Representative and statistical graph for HIF1α in 8 ml medium treated H441 cells cultured in six well plate. ^**^*P* < 0.01, compared with 0 hour (h). n = 4. (B, D) Representative and statistical graph for HIF1α after 300 μM CoCl_2_ treatment. ^*^*P* < 0.05, compared with 0 hour. n = 4. Mann-Whitney U test was used to analyze the difference of the means for significance.
