# Supplementary figures and images for: Airway Basal Cells Mediate Hypoxia-Induced EMT by Increasing Ribosome Biogenesis
Source: Front Pharmacol. 2021 Dec 9;12:783946. doi: 10.3389/fphar.2021.783946 (PMC8696177; doi:10.3389/fphar.2021.783946)

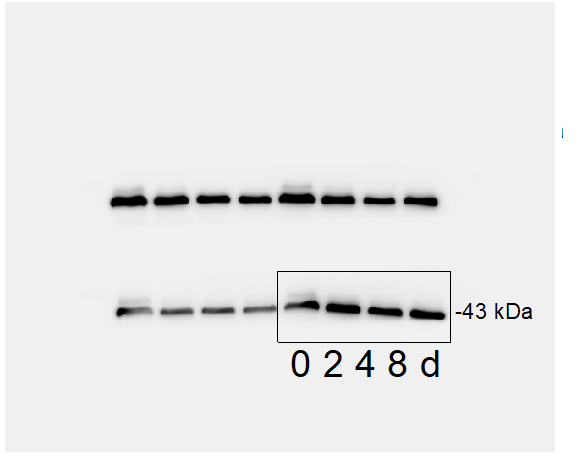

Supplement: Supplementary file 1 [file DataSheet1.ZIP › Supplementary materials/Original source data/Original western blot images/Fig.1/actin for Fig.1E HIF1a days .tif]

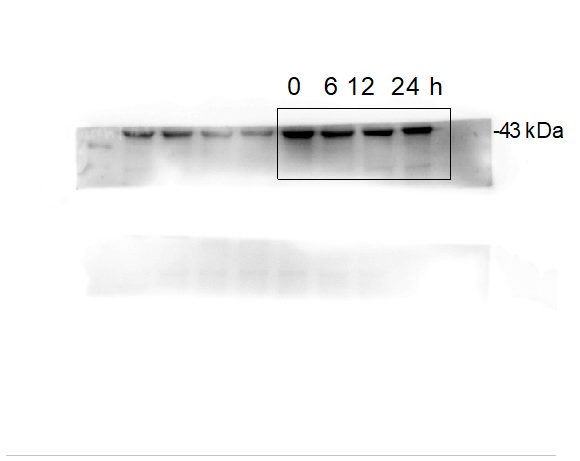

Supplement: Supplementary file 1 [file DataSheet1.ZIP › Supplementary materials/Original source data/Original western blot images/Fig.1/actin for Fig.1E HIF1a hours .tif]

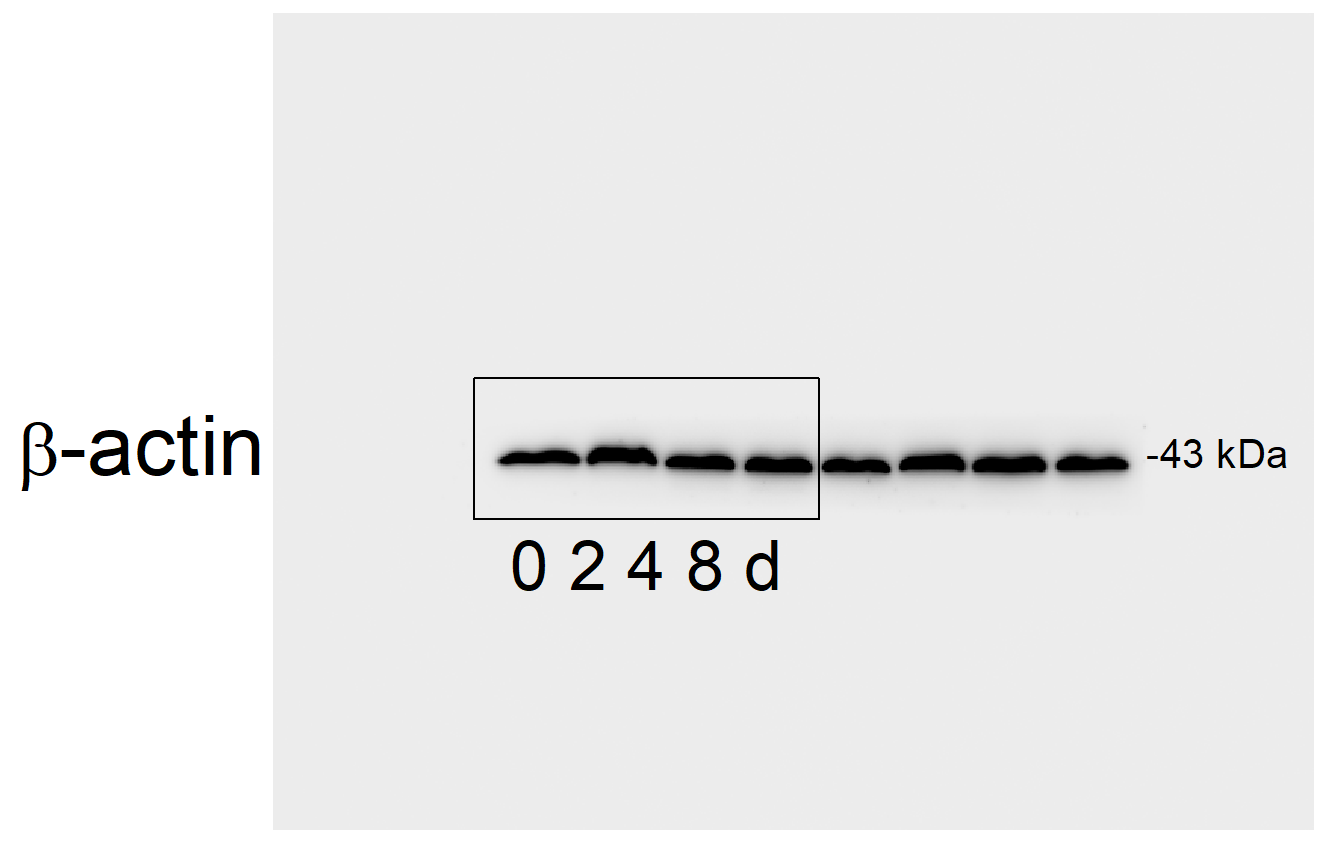

Supplement: Supplementary file 1 [file DataSheet1.ZIP › Supplementary materials/Original source data/Original western blot images/Fig.1/actin for Fig.1E HIF2a.tif]

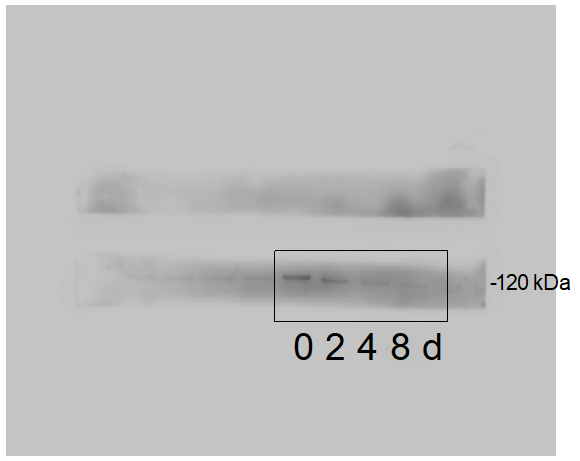

Supplement: Supplementary file 1 [file DataSheet1.ZIP › Supplementary materials/Original source data/Original western blot images/Fig.1/Fig.1E HIF1a days.tif]

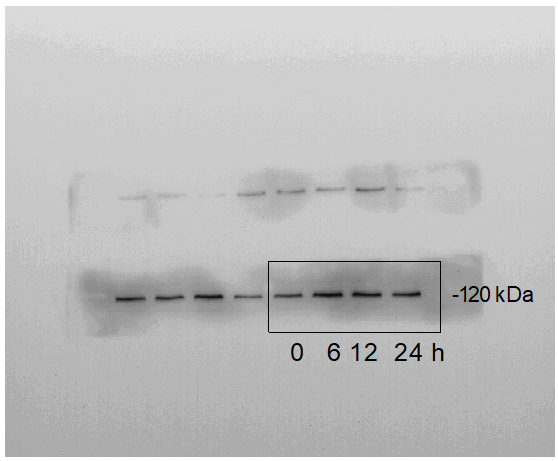

Supplement: Supplementary file 1 [file DataSheet1.ZIP › Supplementary materials/Original source data/Original western blot images/Fig.1/Fig.1E HIF1a hours.tif]

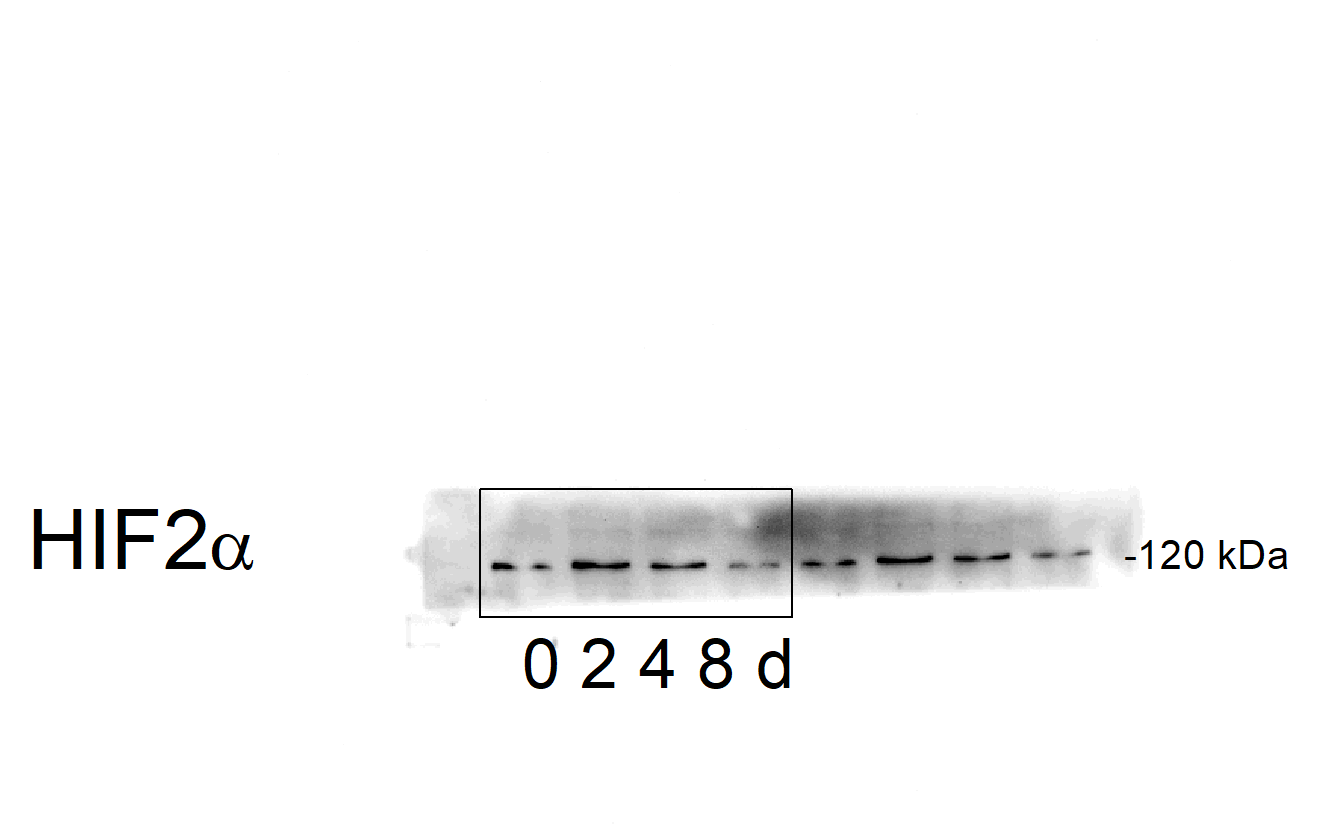

Supplement: Supplementary file 1 [file DataSheet1.ZIP › Supplementary materials/Original source data/Original western blot images/Fig.1/Fig.1E HIF2a.tif]

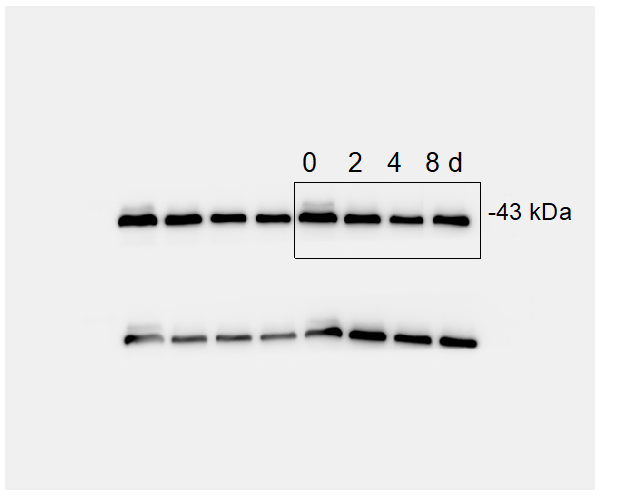

Supplement: Supplementary file 1 [file DataSheet1.ZIP › Supplementary materials/Original source data/Original western blot images/Fig.2/Fig.2B actin.tif]

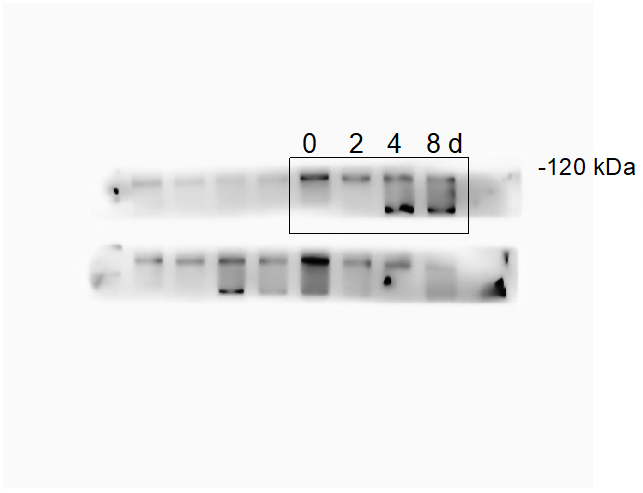

Supplement: Supplementary file 1 [file DataSheet1.ZIP › Supplementary materials/Original source data/Original western blot images/Fig.2/Fig.2B Ecad.tif]

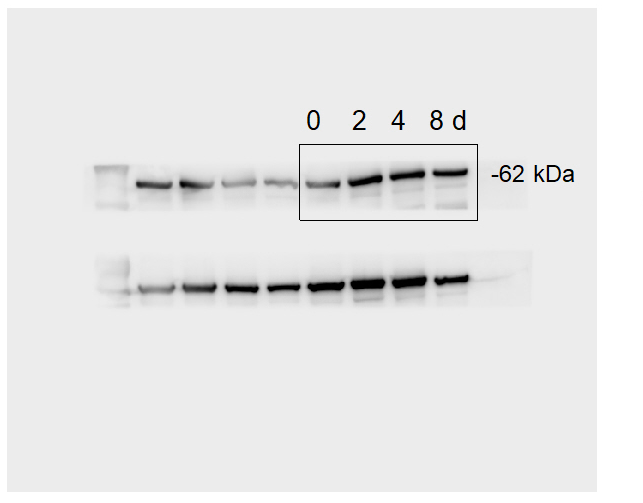

Supplement: Supplementary file 1 [file DataSheet1.ZIP › Supplementary materials/Original source data/Original western blot images/Fig.2/Fig.2B krt5.tif]

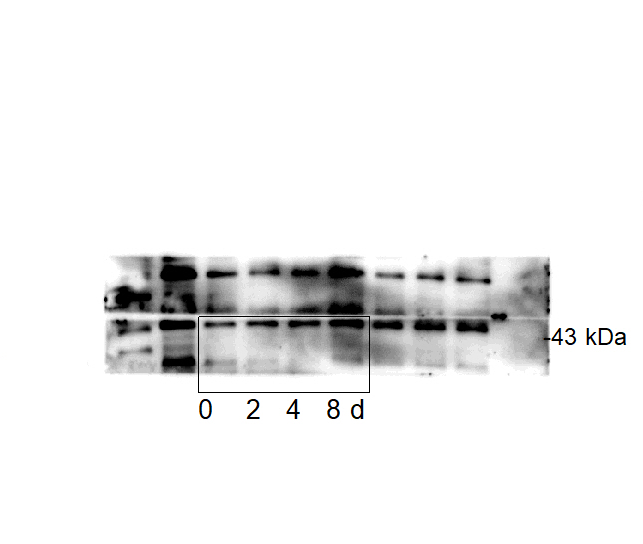

Supplement: Supplementary file 1 [file DataSheet1.ZIP › Supplementary materials/Original source data/Original western blot images/Fig.2/Fig.2B SMA.tif]

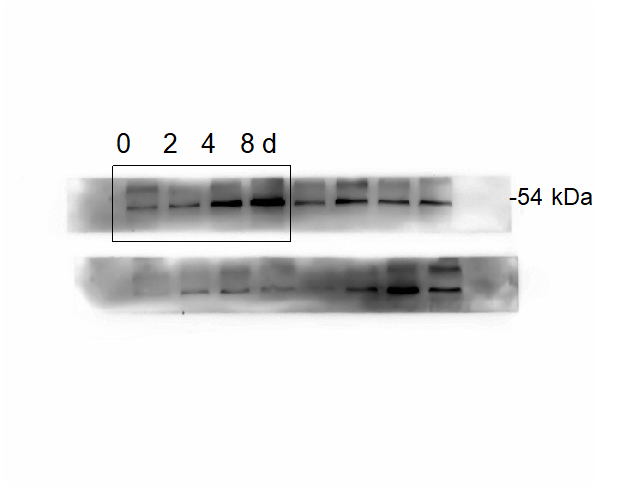

Supplement: Supplementary file 1 [file DataSheet1.ZIP › Supplementary materials/Original source data/Original western blot images/Fig.2/Fig.2B vim.tif]

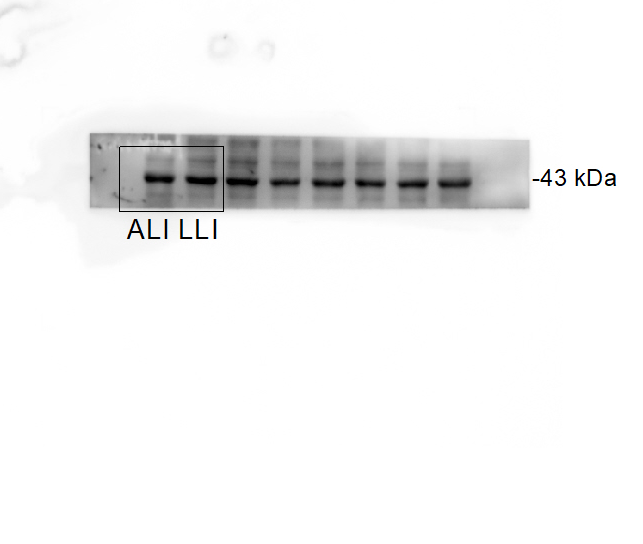

Supplement: Supplementary file 1 [file DataSheet1.ZIP › Supplementary materials/Original source data/Original western blot images/Fig.4/actin for Fig.4F c-myc.tif]

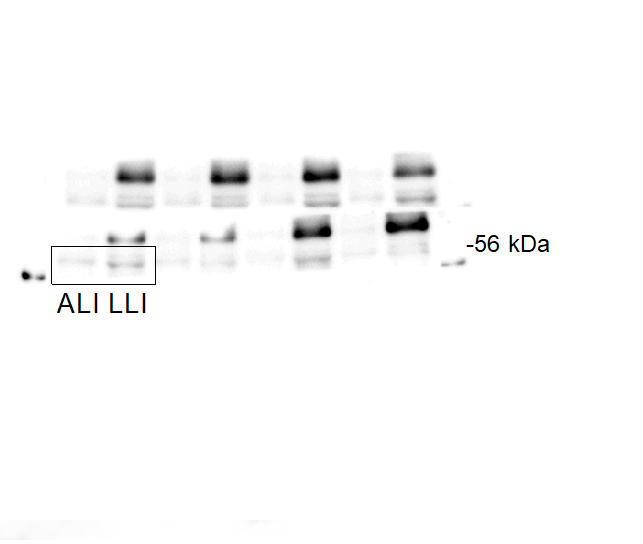

Supplement: Supplementary file 1 [file DataSheet1.ZIP › Supplementary materials/Original source data/Original western blot images/Fig.4/Fig.4F c-myc.tif]

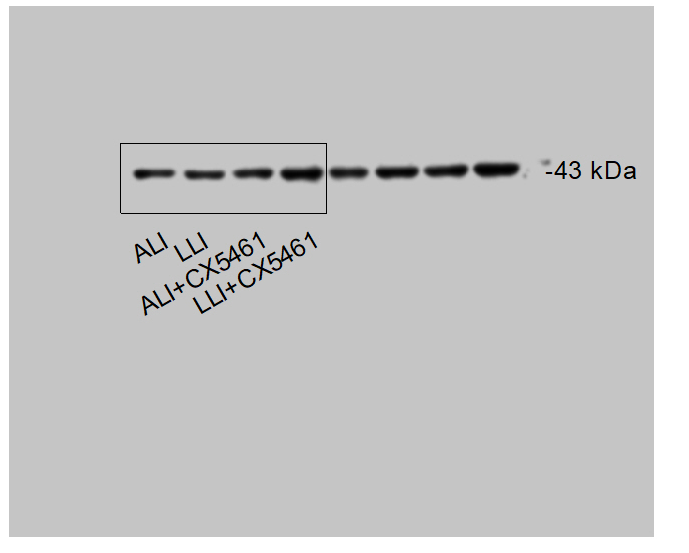

Supplement: Supplementary file 1 [file DataSheet1.ZIP › Supplementary materials/Original source data/Original western blot images/Fig.6/[╘¡╩╝╬─╝■]/Fig.6C actin.tif]

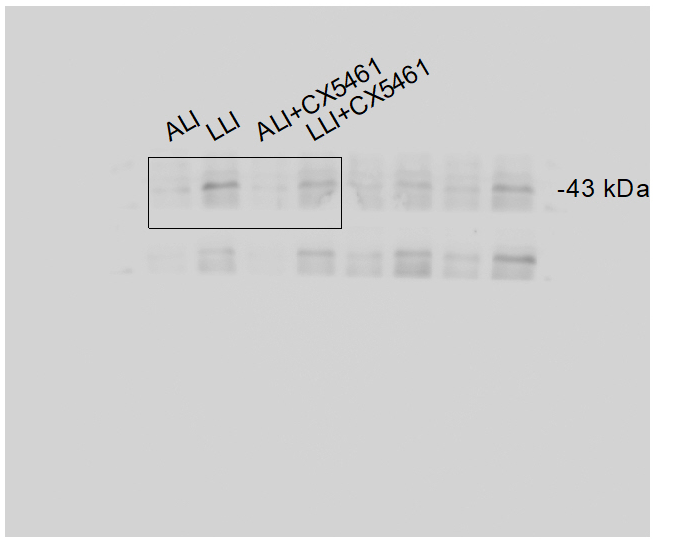

Supplement: Supplementary file 1 [file DataSheet1.ZIP › Supplementary materials/Original source data/Original western blot images/Fig.6/[╘¡╩╝╬─╝■]/Fig.6C SMA.tif]

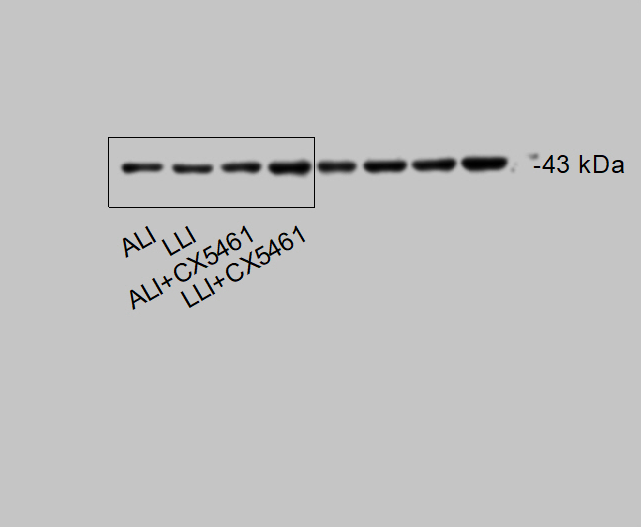

Supplement: Supplementary file 1 [file DataSheet1.ZIP › Supplementary materials/Original source data/Original western blot images/Fig.6/Fig.6C actin.tif]

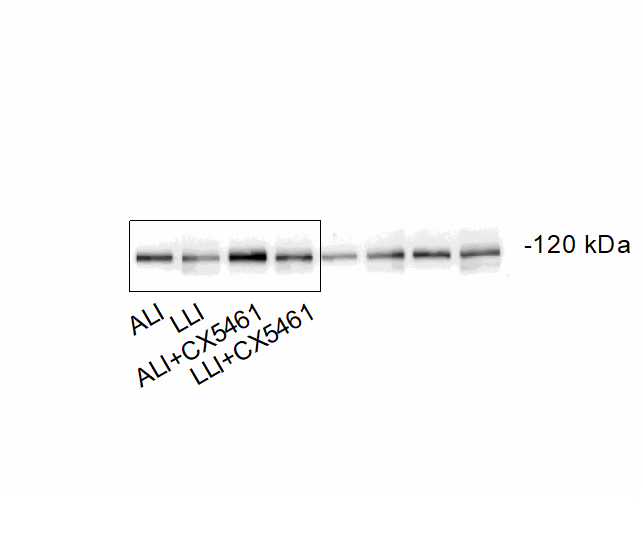

Supplement: Supplementary file 1 [file DataSheet1.ZIP › Supplementary materials/Original source data/Original western blot images/Fig.6/Fig.6C Ecad.tif]

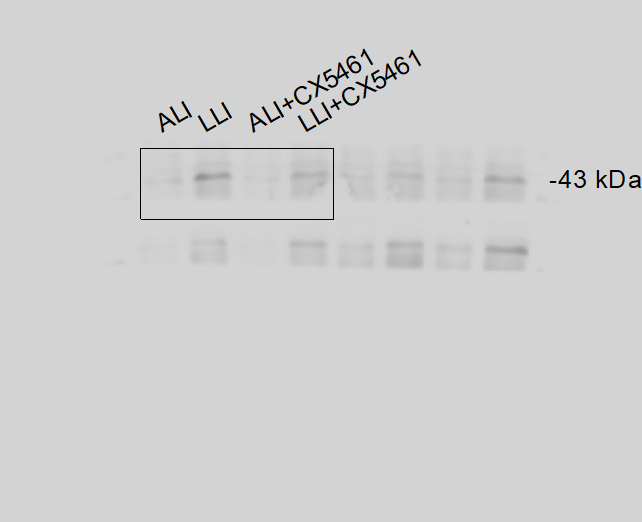

Supplement: Supplementary file 1 [file DataSheet1.ZIP › Supplementary materials/Original source data/Original western blot images/Fig.6/Fig.6C SMA.tif]

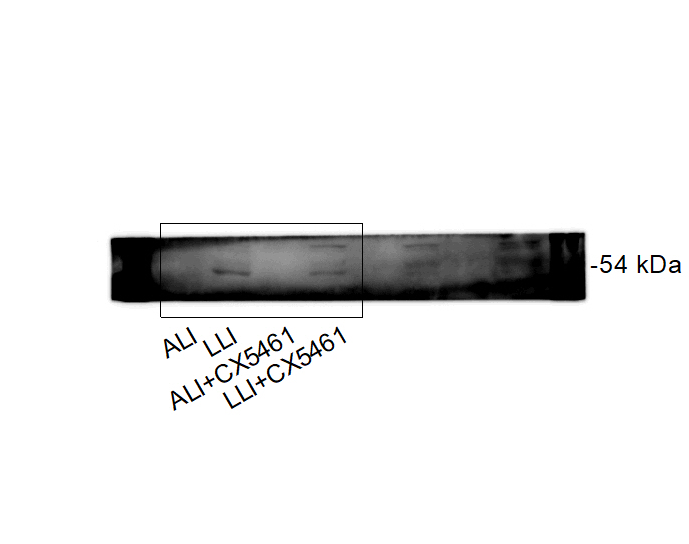

Supplement: Supplementary file 1 [file DataSheet1.ZIP › Supplementary materials/Original source data/Original western blot images/Fig.6/Fig.6C Vim.tif]

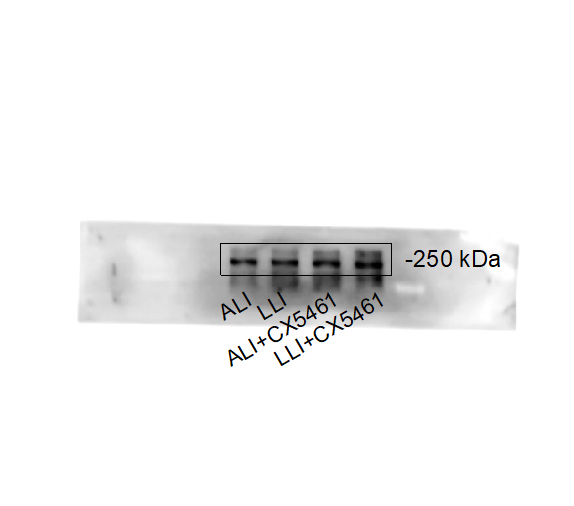

Supplement: Supplementary file 1 [file DataSheet1.ZIP › Supplementary materials/Original source data/Original western blot images/Fig.7/Fig.7A mTOR.tif]

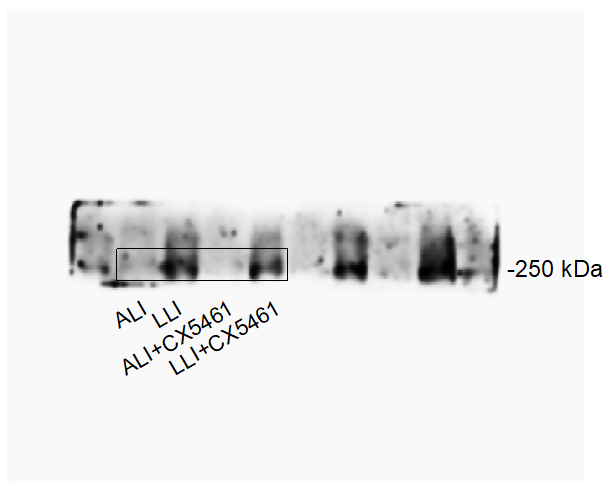

Supplement: Supplementary file 1 [file DataSheet1.ZIP › Supplementary materials/Original source data/Original western blot images/Fig.7/Fig.7A p-mTOR.tif]

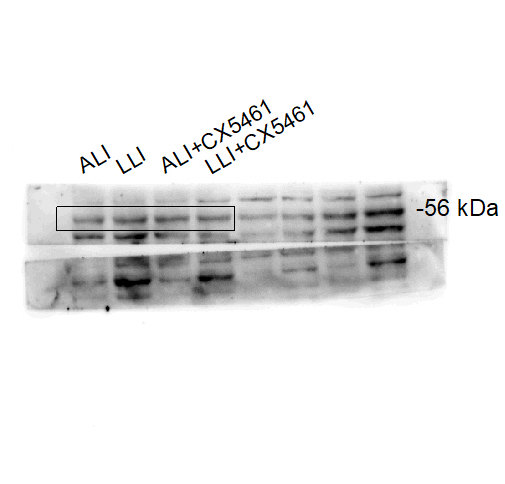

Supplement: Supplementary file 1 [file DataSheet1.ZIP › Supplementary materials/Original source data/Original western blot images/Fig.7/Fig.7B AKT.tif]

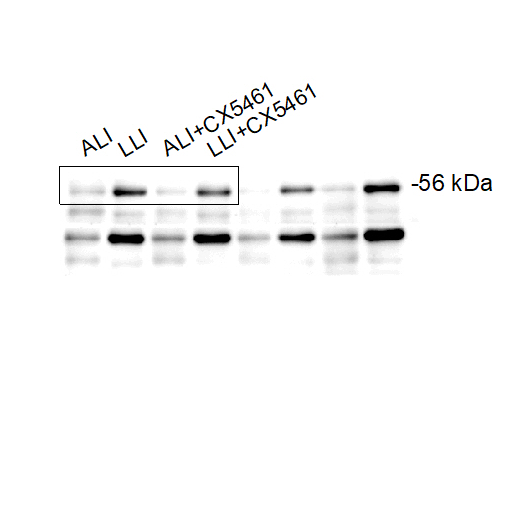

Supplement: Supplementary file 1 [file DataSheet1.ZIP › Supplementary materials/Original source data/Original western blot images/Fig.7/Fig.7B p-AKT.tif]
